# Hybrid Polypropylene Biocomposites Reinforced with Short Man-Made Cellulose Fibres and Softwood Flour—Optimisation of Properties Using Response Surface Methodology

**DOI:** 10.3390/ma18061239

**Published:** 2025-03-11

**Authors:** Piotr Franciszczak, Andrejs Kovalovs, Magdalena Kwiatkowska

**Affiliations:** 1 Department of Machines Construction and Materials, Faculty of Marine Engineering, Maritime University of Szczecin, Willowa Street 2, 71-650 Szczecin, Poland; 2 Institute of High-Performance Materials and Structures, Faculty of Civil and Mechanical Engineering, Riga Technical University, Kipsalas Street 6A, LV-1048 Riga, Latvia; andrejs.kovalovs@rtu.lv; 3 Department of Materials Technology, Faculty of Mechanical Engineering and Mechatronics, West Pomeranian University of Technology Szczecin, Piastow Av. 19, 70-310 Szczecin, Poland

**Keywords:** polypropylene matrix, wood–plastic composites, man-made cellulose, injection moulding, hybrid effect, mechanical properties

## Abstract

Wood–polymer composites and composites reinforced with natural and man-made cellulose fibres are being extensively used in the automotive and building industries. The main shortcoming of the former is their low-impact resistance and brittleness. The relatively high cost of natural and cellulose fibres is the limitation of the latter. This research uses a hybrid combination of wood flour and short man-made cellulose fibres to develop polypropylene composites for injection moulding that excel in mechanical characteristics and have low material cost. Both reinforcements are of wood origin. The synergistic hybrid effect of this combination of reinforcements helps to achieve their mechanical performance superior to that of wood–polymer composites at preserved low cost. The proposed Response Surface Methodology enables the calculation of necessary weight fractions of two reinforcements to achieve desired mechanical properties like strength, tensile, flexural modulus, and impact resistance.

## 1. Introduction

Wood–polymer composites (WPCs) and natural fibre composites (NFCs) are used in building and automotive industries [1,2]. They can be processed by extrusion, compression moulding or injection moulding which makes them a suitable material for high-output manufacturing [3,4]. Both types of composites are reinforced with lignocellulose fibres or lignocellulose particles of fibrous shape [5].

Softwood flour is relatively cheap; therefore, WPCs found their place in the building industry while NFCs are predominantly applied in interior car parts owing to better mechanical properties [6,7]. The particular type of short-fibre biocomposites that are akin to NFCs are composites reinforced with man-made cellulose fibres [8]. These fibres are spun from previously dissolved and purified cellulose obtained from wood pulp—this process has been used in the industry for decades. One of the technical grades of man-made cellulose fibres is manufactured in the rayon viscose process [9]. Rayon fibres have even better and more uniform mechanical properties and provide higher toughness than natural fibres but are also more costly [10].

The use of hybrid reinforcement as a cost-saving measure and a means for tailoring mechanical properties has been applied in the composite industry for a long time when talc, mica, and other mineral fillers [11] were added to glass fibre reinforcement in polypropylene composites to achieve higher stiffness, which resulted also in higher flexural strength [12]. Currently, a widespread hybrid combination of glass and carbon fibre reinforcements is used in the form of textiles and short fibres in thermoset and thermoplastic composites [13,14]. Beyond the fact that high-performance yet costly carbon fibres provide a great increase in strength and stiffness, cheaper glass fibres provide high toughness [15]. Similarly, in the case of polypropylene composites reinforced with softwood flour, their mechanical properties can be significantly increased by the addition of short man-made cellulose fibres [16].

The process of reinforcement using a combination of cellulose microfibres and short man-made cellulose fibres is more effective than hybridisation of short glass fibres with cellulose microfibres owing to their less brittle characteristics in opposite to glass fibres [17]. The hybrid combination of wood flour and short man-made cellulose fibres can also be used to improve the brittle characteristics of WPCs instead of using elastomer impact modifiers, whose incorporation indeed improves greatly their impact resistance, but it also reduces WPC’s strength and modulus [18]. The use of additional short man-made cellulose fibres moderately increases impact resistance, which comes with an even higher increase in composite strength and Young’s modulus.

The following paper presents the application of Response Surface Methodology for the calculation of optimal weight fractions of softwood flour and short man-made cellulose fibres for achieving desired composite mechanical properties [19,20]. The obtained response surfaces in the form of 3D graphs are an easy engineering tool that can be applied for material cost reduction and tailoring the desired properties by choosing the proper weight fraction of softwood flour and short man-made cellulose fibres determined in the design phase of a product.

## 2. Materials and Methods

### 2.1. Materials

Polypropylene (PP) homopolymer HP400R, Bassel-Orlen, Płock, Poland, MFR = 25 and MVR = 34 (230 °C/2.16 Kg) was used as a matrix. TP Licocene PP MA 7452, Clariant, Muttenz, Switzerland, which is a maleic acid anhydride grafted PP wax (MAH-g-PP) was used as a compatibiliser between polypropylene matrix and man-made cellulose (Rayon) fibres and lignocellulose (softwood) reinforcement [21,22]. This effective compatibiliser is widely used in PP-based composites with reinforcement having hydroxyl groups [23]. Both PP and compatibiliser granulates were mixed together prior to pouring into the feeder’s chamber. Compatibiliser weight content was set in a way that provided a constant ratio of its volume to the volume of reinforcement. It was ~2.2% for all composites except for these with 10 and 20 wt.% of reinforcement for which the ratio was 5 and 3%, respectively, to ensure obtaining of compatibilised fibre-matrix interphase in case the retained compatibiliser has been partially left unreacted due to greater volume of matrix. The short man-made cellulose rayon fibres (Cordenka) and softwood flour (Weho 500) were used as reinforcement [24,25].

Cordenka 610 F Super 2—this high-tenacity rayon viscose tire grade yarn is manufactured by Cordenka, Frankfurt am Main, Germany. The applied yarn has a linear density (nominal) dtex = 1840 × 2 and has 1000 filaments [23,25]. Its breaking force is 89.6 N. It has a density of 1.627 g/ccm. The diameter of each single fibre is 17–18 mm [24]. The yarn was cut to lengths ranging from 1.7–2 mm by EKOTEX/Namysłów, Poland to ensure good dry mixing with short rayon fibres and trouble-free dosing by gravimetric feeders during compounding. It was also tested in preliminary evaluation that higher 3 and 4 mm lengths of short rayon fibres did not significantly improve the mechanical performance of manufactured composites having volume fractions of fibres > 30 wt.% while the feeding was hindered.

The softwood flour Weho 500 is manufactured by Jeluwerk, Aulendorf, Germany. This is a fine grade of softwood flour used in the wood–plastic composite industry. It is a mix of microfibres and particles obtained from European spruce (*Picea abies*) and silver fir (*Abies alba*). Its main component is lignocellulose. Its appearance is a light-yellow powder of ~130 g/L bulk density and density of ~1.48 g/ccm according to the datasheet.

### 2.2. Methods

#### 2.2.1. Manufacturing of Composites

**Figure 1:**
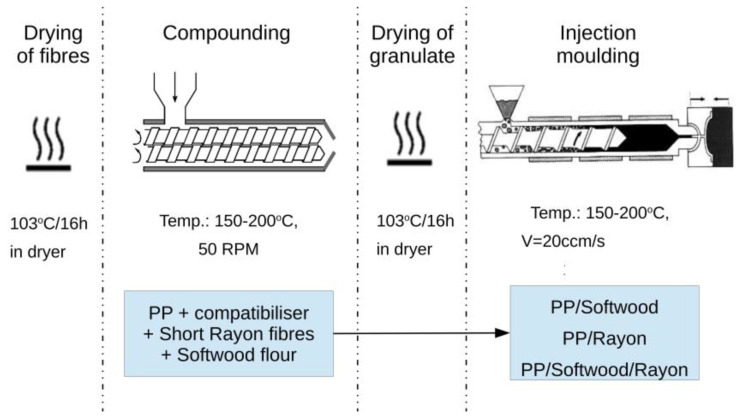
Production process of short-fibre PP composites.

The production process of short-fibre polypropylene composites is presented in Figure 1. Both short rayon fibres and softwood flour were dried in a fanned drying oven SLW115, (POL-EKO, Kędzierzyn-Koźle, Poland) for ~16 h at 103 °C to achieve material moisture <0.1 wt%. By compounding a composite with hybrid reinforcement, the short fibres were dry-mixed with softwood flour in a closed bucket (using a manual stirrer) prior to pouring them into the chamber of the gravimetric feeder. Due to the highly hygroscopic nature of cellulose, their exposure to ambient air was reduced to a minimum and random control at the end of feeding, which always showed results below 0.2 wt.% for different batches. The short rayon fibres and softwood flour were fed into a compounder from twin-screw gravimetric feeder DDSR20 (Brabender, Duisburg, Germany), while another single screw gravimetric feeder DSR28 (Brabender, Duisburg, Germany) was used to feed the dry-mixed PP and compatibiliser granulates (same method as for softwood and short fibres) into the compounder counter-rotating, tight intermeshing twin-screw extruder Laborextruder LSM30 (Leistritz, Nuremberg, Germany). The L/D = 23 and D = 34 mm. The composite compound extrudates were cooled in a water tank and pelletised. To ensure easy processing, yet to reduce the decomposition of lignin and cellulose, the barrel zones of the compounder were set to temperatures from 150 °C to 200 °C (feed zone to nozzle) with 5 °C increment on each zone, according to a well-known practice applied in the processing of wood–plastic composites [26]. The compounding was conducted at 50 RPM. The output was set to 1.5–2.5 kg/h, depending on the amount of fibres and softwood fed into the compounder. This difference is explained in the discussion section.

The type A (dog-bone) and type B (flexural bar) test specimens compliant with standards EN ISO 294-1 and Type D2 60 × 60 × 2 mm plates for impact testing compliant with EN ISO 294-3 were injection moulded according to EN ISO 178 using an ALLROUNDER 270 S 350-100 (ARBURG, Lossburg, Germany). The clamping force is 350 kN, the screw diameter is 25 mm, and L/D = 20. Granulates of composite compounds were dried prior to injection moulding in the same way as rayon viscose fibres and cellulose microfibres. The barrel temperatures were 160–200 °C from the feed zone to the nozzle with a 10 °C increment for each heating zone. The mould temperature was set to 40 °C for batches < 70 wt.% and to 70 °C for batches ≥ 70 wt.%. Back pressure by dosing was set to 50 bars. The circumferential screw speed by dosing was set to 120 mm/s. The injection volume flow was set to 20 ccm/s, which resulted in actual injection pressures ranging from 400 to 2000 bars, depending on volume fraction and type of reinforcement in each manufactured batch. The holding pressure was set to increase from 200 to 1000 bars during 15–45 s to effectively allow fill up of shrinking molten compound during the cooling stage (longer increase times of after pressure for lower filling ratios of reinforcement to avoid mould overflow and shorter times for higher filling ratios of reinforcement to allow fill up of shrinkage before spruce solidifies). The holding pressure time together with the cooling time took 30–60 s (the higher the weight fraction of reinforcement the higher the injection pressures, yet also the shorter cooling was needed). It was evaluated that, at these processing conditions depending on batch, it took 2.5–3 min for the material to go from the feed section to be ejected as an injection moulded part.

#### 2.2.2. Evaluation of Composite Properties

The density of manufactured composites was measured on flexural bars using the Archimedes method at room temperature on a high accuracy scale type AS (Radwag, Poland), according to standard EN ISO 1183. Samples were measured in an ethanol medium. Weighing was repeated three times for each of ten different samples measured for each type of material and the average of the measured values was taken as a result.

The theoretical density of composites was calculated according to the following formula:

(1) $$\rho_{T\;comp.}=\frac{100}{\frac{{Wt.\%}_{PP}}{\rho_{PP}}+\frac{{Wt.\%}_{Rayon}}{\rho_{Rayon}}+\frac{{Wt.\%}_{Softwood}}{\rho_{Softwood}}}$$

Composites’ porosity (void fraction) was calculated according to the following formula:

(2) $$V_{vol.\%}=1-\frac{\rho_{R\;comp.}}{\rho_{T\;comp.}}$$

The volume content of the matrix was calculated according to the following formula:

(3) $$M_{vol.\%}=\frac{Wt.{\%}_{PP}{\;\cdot \;\rho }_{Tcomp.}}{{\;\;\rho }_{Rcomp.}\;\cdot \;(1-V_{vol.})}$$

The volume content of softwood flour reinforcement was calculated according to the following formula:

(4) $${Softwood}_{vol.\%}=\frac{{Wt.\%}_{Softwood}\;\cdot \;\rho_{T\;comp.}}{\rho_{R\;comp.}\;\cdot \;\left(1-V_{vol.}\right)}$$

and volume content of short rayon fibre reinforcement was calculated according to the following formula:

(5) $${Rayon}_{vol.\%}=\frac{{Wt.\%}_{Rayon}\;\cdot \;\rho_{T\;comp.}}{\rho_{R\;comp.}\;\cdot \;\left(1-V_{vol.}\right)}$$

where ${Wt.\%}_{pp}$ is the weight content of the polypropylene matrix, ${Wt.\%}_{Rayon}$ is the weight content of rayon fibres, ${Wt.\%}_{Softwood}$ is the weight content of softwood flour, $\rho_{PP}$ is the density of the polypropylene matrix, $\rho_{Rayon}$ is the density of Rayon fibres, $\rho_{Softwood}$ is the measured density of softwood flour, and $\rho_{Rcomp.}$ is the measured density of the composite.

Tensile tests according to standard EN ISO 527 and flexural tests according to EN ISO 178 (3-point bending) were applied to evaluate the tensile and flexural performance of manufactured composites. Tensile testing was carried out using a universal testing machine of Instron Electropulse E3000 (Norwood, MA, USA) equipped with video extensometer Imetrum Video Strain Gauge system (Bristol, UK) equipped with IM-CAM-036 camera and IM-LENS-GP006 lens with a local gauge length of 50 mm; 3-point bending was conducted using the AGS-X universal testing machine of Schimadzu (Kyoto, Japan). In all cases, 1 mm/min was the testing speed. All mechanical tests including notched Izod and drop-weight testing were carried out at room temperature 23 ± 1 °C and 50% ± 10 relative humidity at least 2 weeks after injection moulding of samples used for these measurements. Properties were evaluated by measuring 10 specimens of each manufactured composite.

The Izod EN ISO 180/A method was applied to test the notched impact strength of manufactured composites. For this purpose, the Typ B5102 apparatus of Zwick Z100 (Zwick GmbH, Ulm, Germany) was used and a dedicated notching machine was used to make A-notch in samples. The pendulum velocity at impact for this method was 2.9 m/s.

The low-velocity impact tests were performed according to standard EN ISO 6603 and using a Dynatup 9250 HV Impact Tower (Instron, UK). The total mass of the impactor (including a striker and a drop weight framework) was 5.83 kg. The hemispherical type of striker with dia. of 25.4 mm was used. The drop height of 0.39 m gave a kinetic energy of 22 J to the drop-weight system. The corresponding velocity of the striker at the moment of impact was 2.61 m/s. The specimens were clamped between clamping rings of 40 mm diameter.

#### 2.2.3. Design of Experiment

The experiment was designed using the full factorial design (FFD). This procedure is carried out when all variables are changed in experimental runs and includes all possible combinations of levels for all factors [27,28]. In this study, FFD consisted of $m^{k}$ runs, where *k* is the number of factors and *m* is the number of levels for each reviewed variable. The design of the experiment consisted of *k* = 2 design factors: short man-made cellulose fibres (Cordenka) and softwood flour (Weho 500) and *m* = 5 weight fractions in the manufactured composites. Thus, 25 experimental runs for two factors at five levels have been performed which equalled to manufacturing of 25 different composites. The minimum and maximum levels for design factors are listed in Table 1.

The mathematical relationship between corresponding factors and their responses can be expressed by different approximation functions using experimental results obtained in the points of design of the developed experiment. In the presented study, regression equations were modelled through a polynomial equation of first, second, and third orders comprising linear, quadratic, cubic, and interactive terms:

(6) $$y=b_{0}+\sum_{i=1}^{m}b_{i}x_{i}+\sum_{i=1}^{m}b_{i}x_{i}^{2}+\sum_{i=1}^{m}\sum_{j=1}^{m}b_{ij}x_{i}x_{j}+\sum_{i=1}^{m}b_{iii}x_{i}^{3}+\sum_{i=1}^{m}\sum_{j=1}^{m}\sum_{l=1}^{m}b_{ijl}x_{i}x_{j}$$

where y is the response; $x_{i}$ and $x_{j}$ are the uncoded values of factors; $b_{0}$ is the constant coefficient; $b_{i}$, $b_{ij}$ and $b_{ijl}$ are the linear, quadratic, cubic, and interactive coefficients, respectively; m is the number of the factors.

The experimental results were analysed using EdaOpt v.2.96 software [29]. The adequacy of the polynomial equations was evaluated with the help of an adjusted coefficient of determination $R_{adj}^{2}$, the relative cross-validation error $\sigma_{cross}$, root-mean-square error σ, and relative root-mean-square error $\sigma_{r}$. For regression model validation, the adjusted coefficient of determination $R_{adj}^{2}$ indicates the reliability of a model and has to tend to 1. Root-mean-square error *σ* and its relative value *σ_r_* should be of minimal values. According to the model used by Yorukoglu M. et al. [30], adequacy is considered good when the relative root-mean-square error *σ_r_* is less than 20% and fair until 30%. When relative cross-validation error *σ* cross tends to 100%, the obtained regression model is poor [31]. Thus, all estimates were considered simultaneously to evaluate the accuracy and reliability of the developed regression models.

Evaluation of numerical accuracy in the estimation of regression coefficients was applied to reduce the cost of chosen models by removal of unrequired excess terms from the models. This method includes a leave-one-out methodology to determine the values for each coefficient when each sample is removed from the model. The average value for each coefficient is then determined, along with the corresponding margin of error (*MoE*), when calculating a 95% confidence interval as defined by Equation (7):

(7) $$MoE=1.96\frac{\sigma }{\sqrt{n}}$$

where σ is the standard deviation of the constant, and n is the number of samples (total 25).

When the sum of the average value and the margin of error in the possible constant range is both positive and negative, the term is removed from the predictive model and the polynomial equation is recalculated. Red dots visible on the plots represent the averaged values of properties evaluated during measurements of fabricated composites.

## 3. Results and Discussion

### 3.1. Influence of Hybridisation of Reinforcement on Manufacturing

The softwood flour could be easily mixed with short rayon fibres of ~2 mm in length. Filling ratios up to 50 wt.% of a mixture of short rayon fibres and softwood flour were fed smoothly into the compounder. The bridging effect of a mixture of short fibres and softwood flour occurred when the weight fraction was set to >60 wt.%, and bridging was more profound for higher ratios of short fibres in the mixture. For these batches, feeding output was reduced down to 1.5 kg/h for 40/40 wt.% of reinforcement ratio to avoid bridging. Similarly, by injection, moulding batches having >60 wt.% of reinforcement needed significantly higher injection pressures to keep the injection flow set to 20 ccm/s reaching the limit of 2k bars for 40/40 wt.% of reinforcement ratio. This particular batch had samples with visibly rougher surfaces. It was the effect of the hindered flow of molten compound due to the high content of reinforcement.

### 3.2. Results of Experiments Overview

The results of 25 runs with determined densities and calculated volumetric fractions of reinforcement and porosity are reported in Table 2. The two factors tested in this study were short man-made cellulose fibres (Cordenka) and softwood flour (Weho 500).

Tensile modulus (*E_Tens_*), tensile strength (*σ_Tens_*), tensile strain (*ɛ_Tens_*), flexural modulus (*E_Flex_*), flexural strength (*σ_Flex_*), impact strength (*E_IZOD_*), and absorbed energy (*E_Impact_*) are reported in Table 3.

### 3.3. Adequacy of Approximations

Polynomial functions of different orders were obtained from the software EdaOpt and were used to approximate experimental results and estimate the significance of regression models. Table 4 presents the results of first-, second-, and third-order regression models. According to Table 4, tensile modulus can be expressed by the first-order polynomial equation. The result of *σ_cros_* has the lowest value while other estimates have approximately the same values regardless of the order of the equation. However, flexural modulus has shown fair estimates for second-order polynomial equations in comparison with first-order polynomial equations. The second-order equations are applicable also for density, porosity and tensile strain. The data of Table 4 demonstrate that the third-order polynomial equation of tensile, flexural, notched Izod strength, and absorbed energy values were highly significant with a high value of the adjusted coefficient of determination ($R_{adj}^{2}$ > 0.95) and low values of other estimates, respectively.

Using the margin of error (*MoE*) shows that almost all coefficients have significant effects in polynomial equations. Except for notched Izod strength, where cubic terms of softwood flour (*X*_1_*·X*_1_*·X*_1_) are not significant and density, where the quadratic term of short rayon fibres (*X*_2_*·X*_2_) is not significant, the other terms are found to be significant. The regression coefficients included in the regression models developed for further studying the influence of the short rayon fibres and softwood flour on their responses are summarised in Table 5.

### 3.4. Density and Porosity

**Figure 2:**
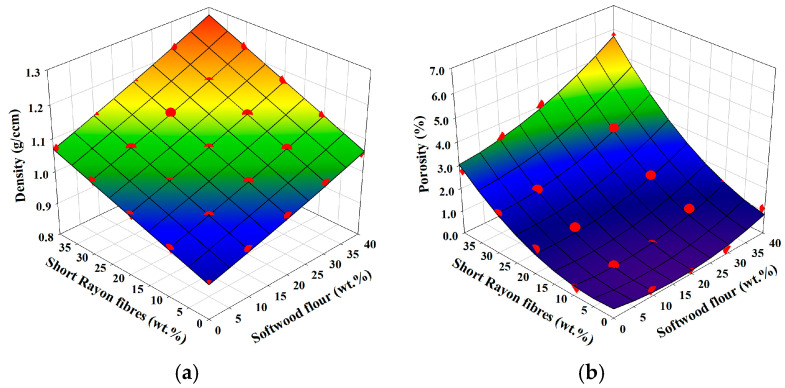
Dependency of composite density and porosity on the weight fractions of reinforcements: (**a**) density; (**b**) porosity. Red dots imposed on calculated response surface represent experimental results.

The response surface plots of composite density and porosity (void content) variables in the function of weight fractions of reinforcements are presented in Figure 2a and Figure 2b, respectively. Owing to similar densities of both types of reinforcements (short rayon fibres and softwood flour) the surface is almost plane-like. However, the measured densities of the manufactured composites diverge from their theoretical values (Table 2). This is caused by the occurrence of voids in the samples of composites with higher reinforcement filling ratios. Voids occurring in injection moulded composite parts can be caused by shrinkage of PP during cooling (PP’s thermal expansion coefficient is 10–21 × 10^−5^/°C in the range of 20–200 °C, which corresponds to ~10% of shrinkage during cooling from 200 °C to room temperature), when the insufficient holding pressure or premature solidification of sprue can lead to inability to fill up the shrinkage [32].

This can be further aggravated by increased molten compound viscosity, which is positively correlated with volume fraction and aspect ratio of reinforcement [33,34]. The next cause is the evaporation of residual moisture from lignicellulose reinforcement and the release of the volatiles due to its slow decomposition at processing temperatures [26]. Finally, reaching the maximum packing fraction of reinforcement volume prevents the formation of a continuous matrix phase in the composite and leads to a rapid increase in porosity [35,36].

It can be seen in Table 2 and Figure 2b that porosity is higher in composites reinforced with short rayon fibres than with their counterparts reinforced with softwood flour. This is due to the lower aspect ratio of the softwood flour reinforcement, which does not increase the viscosity of molten compound as much as short rayon fibre reinforcement. Porosity decreases the effective volume of the composite, which in turn decreases its strength and modulus. Moreover, at a higher void fraction, the reinforcement–matrix bonding area is reduced, which induces crack formation in these places during the straining of the composite [37]. Nevertheless, the processing technique of injection moulding and thorough drying of composite material prior to processing resulted in negligible amounts of voids in most of the manufactured composites, except for the ones with the highest fractions of reinforcement. Results of void content of composites reinforced only with short rayon fibres are in accord with earlier research performed by Franciszczak et al. [17], while, for PP/Softwood/Rayon 30/40/30 and 30/30/40 wt.%, the results are similar to those of Madesen et al. [36] obtained for PP reinforced with unidirectional flax fibres manufactured using the hot compacting method.

### 3.5. Tensile Properties

**Figure 3:**
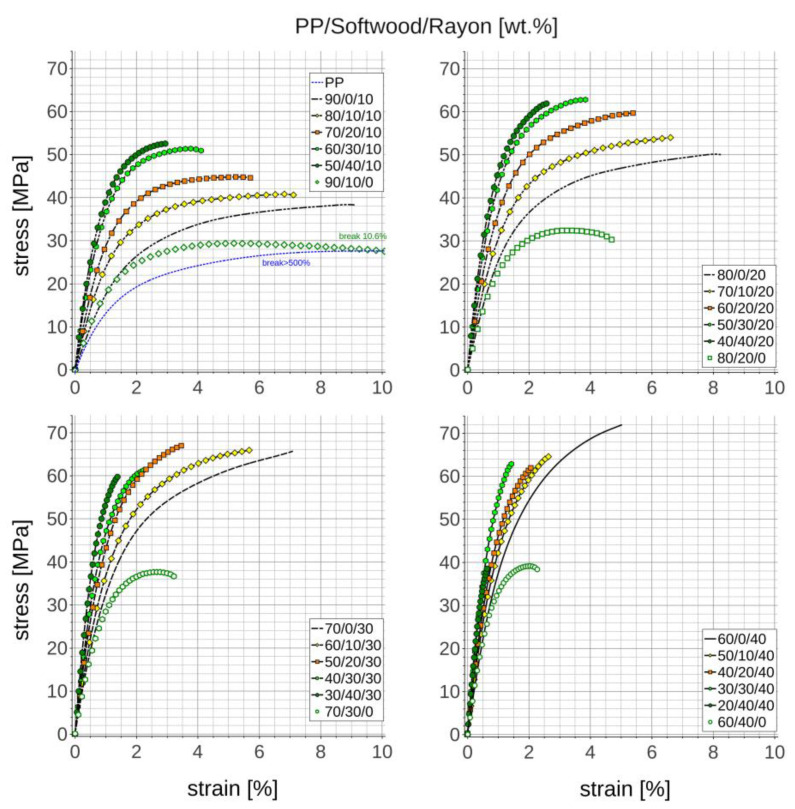
Tensile stress–strain characteristics of manufactured composites.

The tensile stress–strain characteristics of manufactured composites are presented in Figure 3. It shows that although softwood reduces strain to break to a greater extent than short man-made cellulose fibres it increases the composite’s tensile modulus with similar efficiency. Apart from that, the hybrid composites based on 20 and 30 wt.% of short rayon fibres combined with 20–30 wt.% of softwood flour provide reasonable strength on the level of PP composite reinforced with the highest 40 wt.% fraction of short rayon fibres.

**Figure 4:**
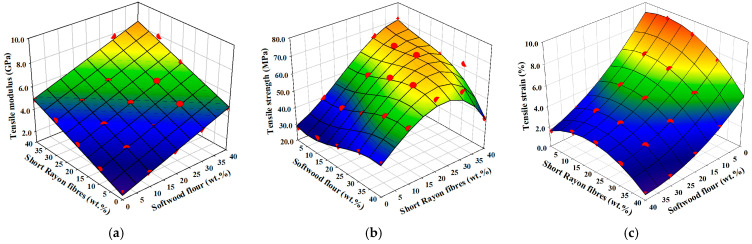
Dependency of tensile properties on the weight fractions of reinforcements: (**a**) tensile modulus; (**b**) tensile strength; (**c**) tensile strain. Red dots imposed on calculated response surface represent experimental results.

The relation between the tensile modulus of the manufactured composites and the weight fractions of their reinforcements is presented in Figure 4a. It can be seen that every rise in the content of short rayon fibres by 10 wt.% results in a 50–60% increase in tensile modulus with respect to the native PP matrix, while every rise in the content of softwood flour by 10 wt.% results in 40–50% increase in modulus.

The important factors, which are attributed to the increase in composite tensile modulus, are the tensile modulus of reinforcement, its volume fraction, aspect ratio and orientation [38,39,40].

Weho500 softwood flour contains 25–30% of lignin, which binds the wood cell walls and provides stiffness [41,42]. Man-made cellulose fibres in turn lack the incrustation by this compound, which is removed from dissolved wood pulp in the rayon viscose process [9]. For this reason, the lower aspect ratio of softwood flour fibrous particles is compensated by their higher stiffness—this is the reason both types of reinforcement give comparable increases in tensile modulus in manufactured composites.

The dependence of the weight fractions of reinforcements in manufactured composites on their tensile strength is illustrated in Figure 4b. The increase in tensile strength is significantly higher for composites reinforced with short rayon fibres than for softwood flour, which is due to the higher strength and aspect ratio of rayon fibres than softwood flour fibrous filler [16]. There is a distinct logistic growth of strength for PP reinforced with short rayon fibres. The increase is limited by a horizontal asymptote placed slightly above 40 wt.%. This limit is caused by the shortening of fibres during processing (compounding and injection moulding), which reduces their effective stress transfer length. The higher the shear viscosity, the more increased shortening is, and shear viscosity is higher by increasing the weight fraction of fibres in the matrix [33]. This loss of performance for composites reinforced with 40 wt.% of short rayon fibres and additional weight fraction of softwood flour > 10 wt.% is also enhanced by the increase in their porosity (Figure 2b). Moreover, exceeding a specific volume of reinforcement for which fillers are agglomerating instead of being separated from each other in the matrix leads to the development of areas which are prone to cracking under load, which also results in composite failure at lower stress and strain [42,43,44]. For these reasons, an additional weight fraction of softwood flour added to composites with higher weight fractions of short rayon fibres did not give any increase in tensile strength but instead led to its decrease, leading to an explicit slump at 40/40 wt.% of reinforcements. However, hybridisation can be very beneficial if low weight fraction of short man-made cellulose fibres is supplemented with additional softwood flour; e.g., the PP/softwood flour/man-made cellulose fibres 60/30/10 wt.% have the same tensile strength as PP/man-made cellulose fibres 70/30 wt.% while having tensile modulus higher by 0.7 GPa. This phenomenon can be attributed to synergistic effects noticed by Franciszczak et al. in similar composite systems [16,17].

The response surface plot of tensile strain (at max. stress) variable in function of weight fractions of reinforcements is presented in Figure 4c. From the graph, the tensile strain at max. stress is reduced by softwood flour to a greater extent than short rayon fibres. This can be attributed to the higher average aspect ratio of rayon fibres [16,17] and their higher ductility due to the lack of rigid lignin compound [9,41,42].

### 3.6. Flexural Properties

**Figure 5:**
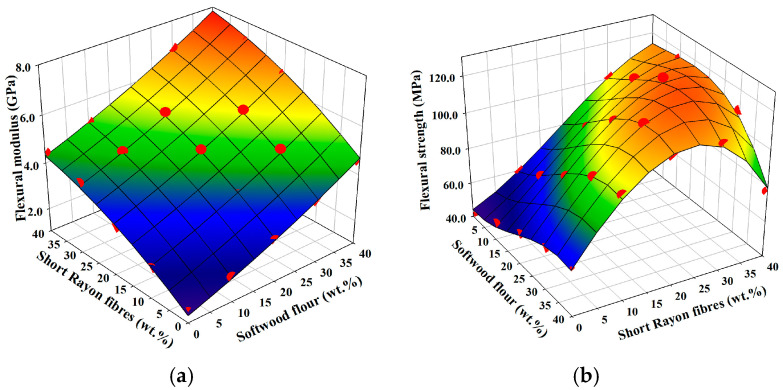
Dependency of flexural properties on the weight fractions of reinforcements: (**a**) flexural modulus; (**b**) strength. Red dots imposed on calculated response surface represent experimental results.

The response surface plot for flexural modulus is presented in Figure 5a. It is also plain-like as in the case of tensile modulus. Generally, short rayon fibres give a similar increase in flexural modulus as softwood flour because the higher aspect ratio of the former is compensated by the higher stiffness of the latter. Similarly, as in the case of tensile modulus, the highest flexural modulus is obtained for the composite with the highest weight fraction of the reinforcement.

Figure 5b presents a response surface plot for the flexural strength of manufactured composites. It has a more distinct plateau around the area with the highest weight fraction of man-made cellulose (rayon viscose) reinforcement than the response surface plot for tensile strength. Although similar composites with the highest weight fraction of short rayon fibres have the highest flexural strength, there is no such distinct drop of flexural strength by additional incorporation of softwood flour in the hybrid reinforcement as in the case of tensile strength. In fact, there is even a slight rising tendency for every weight fraction of short rayon fibres supplemented with up to 30 wt.% of softwood flour. Hence, the benefit of hybridisation of reinforcement can be clearly seen for PP composites made of 10-30 wt.% of rayon fibres with an additional 10–30 wt.% of softwood flour, where both increases with respect to flexural Young’s modulus and flexural strength can be achieved.

### 3.7. Impact Properties

**Figure 6:**
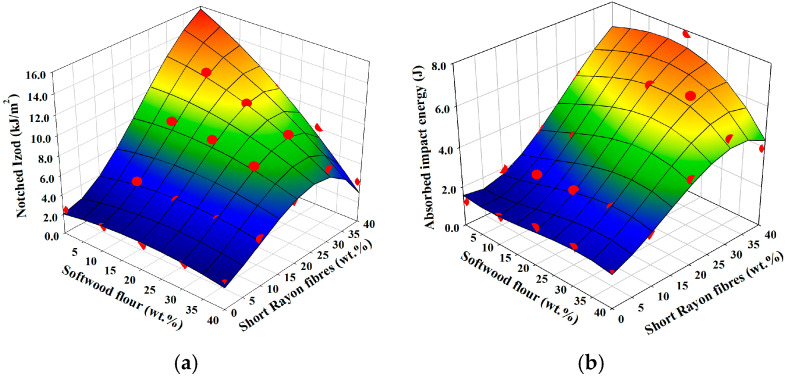
Dependency of impact properties on the weight fractions of reinforcements: (**a**) Izod strength; (**b**) absorbed impact energy in the drop-weight test. Red dots imposed on calculated response surface represent experimental results.

The response surface plot of impact strength evaluated in notched Izod testing regarding weight fractions of reinforcements of manufactured composites is presented in Figure 6a. PP/softwood flour composites have notched Izod strength on a similar level to native PP irrespective of the weight fraction of softwood flour because its fibrous particles have too low an aspect ratio to hinder crack propagation [45]. In turn, the comparably higher aspect ratio of rayon fibres provides an ample increase in notched Izod strength. It is six times higher for PP reinforced with 40 wt.% of short rayon fibres than for native PP or any manufactured composite reinforced only with softwood flour. However, an increasing weight fraction of short rayon fibres gives the logistic growth of notched Izod strength, which tends to be limited by a horizontal asymptote in a similar manner as for tensile strength in these composites. As already mentioned, it is caused by the fact that greater fibre shortening during processing is positively correlated with an increase in fibre weight fraction [16,33]. Also, any additional weight fraction of softwood flour in hybrid reinforcement inflicts extra damage to rayon fibres which leads to the growth of a number of fibre ends, closer fibre–fibre distances in the matrix and agglomerate occurrence which downgrades the ability to arrest the crack propagation upon rapid deformation [46,47]. Moreover, the mentioned decrease in notched Izod strength by additional softwood flour can also be attributed to the increase in composite porosity (Figure 2b). Thus, hybridisation with respect to this property had a negative effect, although, at 10–30 wt.% of short rayon fibres, the effect was negligible for any supplementary softwood flour up to 20 wt.% of its fraction.

From the standpoint of modification of WPC using short rayon fibres, it is clear that the addition of up to 30 wt.% of short rayon fibres increased significantly the Izod strength of WPC, regardless of the content of softwood flour and even the addition of 10 wt.% of short rayon fibres could increase the Izod strength of WPC twofold. Although higher Izod strengths can be achieved by hybridisation with short polyethylene terephthalate (PET) fibres or by the application of elastomer impact modifiers, they both result also with a decrease in composite strength, which is even more profound for the latter [18,48]. Hybridisation with short rayon fibres does lead to a relatively moderate increase in Izod strength; it is however, free from the drawbacks of the discussed approaches. In fact, as presented in Figure 4b and Figure 5b, it leads to an increase in strength.

The response surface plot of energy absorption evaluated in low-velocity drop-weight testing regarding weight fractions of reinforcements of manufactured composites is presented in Figure 6b.

During drop-weight testing, samples are without notches therefore the crack initiation energy is much higher than in notched Izod testing, where notching provides a stress concentration area that promotes a brittle rather than a ductile failure preventing plastic deformation [49]. In drop-weight testing, the striker must first deform the composite plate specimen to initiate the fracture after which another part of the energy is spent to propagate the fracture across the specimen. Therefore, it is substantially different from the mode I fracture initiated in a notch of the cantilever beam during Izod testing, which measures the notch sensitivity of material for energy to fracture [49,50]. For these reasons, the surface response of energies absorbed during impact loading is different than those evaluated in notched Izod. The increase in weight fraction of rayon fibres gives a linear increase in absorbed impact energy and the additional weight fraction of softwood flour has a negligible effect on this property with weight fractions up to 20 wt.% of additional softwood fibrous filler. After exceeding this content, there is a slight decrease in absorbed impact energy for all developed hybrid composites.

## 4. Conclusions

The presented results prove that the hybridisation of short man-made cellulose rayon fibres (Cordenka) with softwood flour (Weho 500) is beneficial in polypropylene matrix composites for injection moulding applications—the addition of softwood flour to short man-made cellulose fibres can improve mechanical properties of developed biocomposites. It means that much more costly short rayon fibres can be replaced with a higher weight fraction of relatively cheap softwood flour without deteriorating the composite’s mechanical performance to save material costs or even to improve its mechanical properties. Softwood flour has also a lower impact on the environment than man-made cellulose making the biocomposites even “greener”. The applied Response Surface Methodology provides a simple tool for the optimisation of proposed hybridisation regarding mechanical properties and the cost of developed composites. The following conclusions on the hybridisation of short rayon fibres with softwood flour in the manufacturing of polypropylene biocomposite can be drawn:

- Tensile modulus is significantly increased by extra weight fractions of softwood flour added to short rayon fibre reinforcement;
- Tensile strength can be slightly increased by adding softwood flour in composites having up to 20 wt.% of short rayon fibres;
- Flexural strength can be significantly increased by adding softwood flour in composites having up to 30 wt.% of short rayon fibres;
- Impact resistance is slightly decreased by extra weight fractions of softwood flour added to short rayon fibre reinforcement and the decrease is more profound with respect to the notch sensitivity of composites;
- Weight fractions of reinforcement >70% hinder the process of injection moulding and significantly deteriorate strength and impact properties.

When the hybridisation is applied from the standpoint of wood–polymer composite modification, it can be concluded that the addition of 10–20 wt.% of short man-made cellulose fibres to softwood flour reinforcement can significantly improve tensile modulus and strength and to a moderate extent can improve the impact resistance of wood–polymer composites. The obtained results prove that the hybridisation of short man-made cellulose fibres with softwood flour can reduce the cost of polypropylene biocomposites for injection moulding applications, making them more competitive in the plastic market. The Response Surface Methodology provides an easy tool to tailor their mechanical performance according to demands by selecting proper weight fractions of two reinforcing components.

## Figures and Tables

**Table 1:** Design factors and their level.

Design Factors: *k* = 2 (Reinforcement Type)	Levels: *m* = 5 (wt.%)				
Short rayon fibres	0	10	20	30	40
Softwood flour	0	10	20	30	40

**Table 2:** Experimental design matrix and observed responses—volume fractions and porosity.

Run	Factor X_1_ ^1^	Factor X_2_ ^2^	Weho 500 (wt.%)	Cordenka (wt.%)	PP Matrix (wt.%)	Real Density (g/ccm)	Std. Dev.	Theoretical Density (g/ccm)	Porosity—Void Content (vol.%)
1	0	0	0	0	100.00	0.906	0.002	-	-
2	0	10	11.24	0	88.76	0.945	0.002	0.9470	0.19
3	0	20	19.98	0	80.02	0.982	0.001	0.9818	−0.02
4	0	30	29.57	0	70.43	1.022	0.001	1.0231	0.11
5	0	40	39.11	0	60.89	1.056	0.019	1.0677	1.11
6	10	0	0	10.59	89.41	0.948	0.011	0.9503	0.29
7	10	10	0	20.44	79.56	0.985	0.001	0.9959	1.08
8	10	20	0	30.29	69.71	1.028	0.008	1.0461	1.73
9	10	30	0	40.22	59.78	1.072	0.002	1.1022	2.76
10	10	40	9.92	9.92	80.17	0.983	0.001	0.9870	0.37
11	20	0	9.92	19.84	70.23	1.025	0.002	1.0368	1.15
12	20	10	10.02	30.07	59.91	1.073	0.002	1.0941	1.96
13	20	20	10.00	39.98	50.02	1.115	0.003	1.1553	3.50
14	20	30	19.84	9.92	70.25	1.027	0.001	1.0303	0.36
15	20	40	19.60	19.60	60.80	1.071	0.002	1.0820	1.00
16	30	0	20.04	30.06	49.90	1.126	0.007	1.1479	1.91
17	30	10	20.00	39.99	40.01	1.165	0.004	1.2155	4.15
18	30	20	30.48	10.16	59.36	1.070	0.001	1.0824	1.11
19	30	30	30.18	20.12	49.70	1.121	0.001	1.1411	1.78
20	30	40	29.87	29.87	40.25	1.168	0.005	1.2049	3.10
21	40	0	30.04	40.06	29.90	1.225	0.007	1.2832	4.54
22	40	10	40.33	10.08	49.59	1.118	0.002	1.1338	1.37
23	40	20	40.40	20.20	39.40	1.175	0.001	1.2017	2.25
24	40	30	40.09	30.07	29.84	1.228	0.003	1.2736	3.60
25	40	40	39.76	39.76	20.47	1.273	0.012	1.3529	5.91

^1^ *X*_1_—softwood flour: Weho 500 (wt%); ^2^ *X*_2_—short man-made cellulose fibres: Cordenka (wt%).

**Table 3:** Experimental design matrix and observed responses—mechanical properties.

Run	Factor X_1_ ^1^	Factor X_2_ ^2^	Tensile Modulus (GPa)	Tensile Strength (MPa)	Tensile Strain (%)	Flexural Modulus (GPa)	Flexural Strength (MPa)	Izod Impact Strength (kJ/m^2^)	Absorbed Energy in Impact (J)
1	0	0	1.55 ± 0.03	27.6 ± 0.1	10.4 ± 0.1	1.46 ± 0.02	42.3 ± 0.4	2.4 ± 0.1	1.2 ± 0.2
2	0	10	2.02 ± 0.13	38.4 ± 4.4	9.1 ± 0.4	2.29 ± 0.04	61.0 ± 0.5	3.8 ± 0.2	2.0 ± 0.5
3	0	20	3.08 ± 0.04	50.1 ± 0.9	8.2 ± 0.1	3.02 ± 0.05	79.4 ± 0.6	6.8 ± 0.4	3.2 ± 0.7
4	0	30	4.15 ± 0.07	65.8 ± 1.0	7.2 ± 0.5	4.02 ± 0.08	102.2 ± 0.8	11.7 ± 0.4	5.1 ± 0.6
5	0	40	4.85 ± 0.12	72.0 ± 1.0	5.0 ± 0.2	4.46 ± 0.10	113.8 ± 1.6	14.2 ± 0.4	6.8 ± 0.6
6	10	0	2.20 ± 0.05	29.4 ± 0.3	5.3 ± 0.1	2.07 ± 0.05	49.6 ± 0.7	2.5 ± 0.2	1.4 ± 0.5
7	10	10	3.10 ± 0.04	40.9 ± 0.7	7.0 ± 0.4	2.89 ± 0.03	69.8 ± 0.6	5.1 ± 0.2	2.6 ± 0.5
8	10	20	3.98 ± 0.08	54.4 ± 0.9	6.8 ± 0.8	3.77 ± 0.05	90.7 ± 0.6	9.3 ± 0.5	3.7 ± 0.5
9	10	30	4.82 ± 0.09	66.1 ± 1.1	5.6 ± 0.6	4.63 ± 0.11	111.1 ± 1.0	12.4 ± 0.6	5.5 ± 0.9
10	10	40	5.62 ± 0.16	64.5 ± 5.0	2.6 ± 0.6	5.07 ± 0.07	116.0 ± 2.2	11.0 ± 0.8	7.0 ± 1.0
11	20	0	2.96 ± 0.04	32.5 ± 0.1	3.3 ± 0.1	2.79 ± 0.11	57.2 ± 1.2	2.5 ± 0.2	1.9 ± 0.4
12	20	10	3.89 ± 0.08	45.1 ± 0.6	5.5 ± 0.4	3.79 ± 0.05	82.4 ± 0.9	4.9 ± 0.3	2.8 ± 0.2
13	20	20	4.89 ± 0.15	59.9 ± 0.8	5.3 ± 0.4	4.82 ± 0.04	105.6 ± 0.7	9.1 ± 0.4	3.7 ± 0.5
14	20	30	5.72 ± 0.09	67.2 ± 2.0	3.5 ± 0.5	5.58 ± 0.10	122.2 ± 1.1	10.8 ± 0.9	6.2 ± 1.2
15	20	40	6.24 ± 0.27	62.1 ± 5.1	2.1 ± 0.4	5.63 ± 0.23	114.3 ± 5.2	10.6 ± 1.2	7.9 ± 0.9
16	30	0	3.82 ± 0.05	37.7 ± 0.2	2.7 ± 0.1	3.60 ± 0.03	63.5 ± 0.5	2.5 ± 0.1	2.0 ± 0.2
17	30	10	4.98 ± 0.10	51.4 ± 0.4	3.8 ± 0.1	4.97 ± 0.04	94.9 ± 0.5	4.8 ± 0.4	2.9 ± 0.9
18	30	20	5.91 ± 0.12	62.8 ± 0.8	3.7 ± 0.1	5.79 ± 0.05	115.8 ± 0.8	8.1 ± 0.5	4.2 ± 0.6
19	30	30	6.42 ± 0.21	61.4 ± 1.9	2.2 ± 0.3	6.13 ± 0.21	117.2 ± 5.7	9.3 ± 1.0	6.5 ± 1.3
20	30	40	7.87 ± 0.25	63.3 ± 1.6	1.4 ± 0.1	6.92 ± 0.25	109.6 ± 6.8	8.2 ± 1.4	6.1 ± 1.5
21	40	0	4.77 ± 0.06	39.2 ± 0.2	2.0 ± 0.1	4.56 ± 0.05	65.2 ± 0.6	2.6 ± 0.1	1.7 ± 0.1
22	40	10	5.63 ± 0.06	52.4 ± 0.8	2.8 ± 0.1	5.70 ± 0.07	98.1 ± 1.0	4.8 ± 0.2	2.6 ± 0.5
23	40	20	6.66 ± 0.14	61.8 ± 1.5	2.6 ± 0.3	6.63 ± 0.28	110.3 ± 6.0	6.3 ± 0.8	4.3 ± 1.4
24	40	30	7.97 ± 0.21	60.0 ± 4.1	1.4 ± 0.2	7.28 ± 0.10	110.0 ± 3.4	7.4 ± 1.1	5.3 ± 1.2
25	40	40	8.13 ± 0.30	38.3 ± 3.1	0.6 ± 0.1	7.76 ± 0.74	77.2 ± 9.4	2.4 ± 1.1	3.9 ± 1.0

^1^ *X*_1_—softwood flour: Weho 500 (wt%); ^2^ *X*_2_—short man-made cellulose fibres: Cordenka (wt%).

**Table 4:** Approximation estimates.

Property	*σ_cross_*, %	$R_{adj}^{2}$	*σ*	*σ_r_*, %
First-order polynomial equation				
*r* *_comp_*	9.74	0.99	0.01	8.68
*V_Vol._*	35.05	0.89	0.50	31.91
*E_Tens_*	11.72	0.99	0.19	10.79
*σ_Tens_*	72.84	0.58	8.59	64.99
*ɛ_Tens_*	48.52	0.80	1.15	44.63
*E_Flex_*	14.94	0.98	0.23	14.02
*σ_Flex_*	67.01	0.67	14.69	60.33
*E_IZOD_*	59.89	0.71	2.21	59.89
*E_Impact_*	44.53	0.84	0.78	39.97
Second-order polynomial equation				
*r* *_comp_*	4.94	0.99	0.01	3.76
*V_Vol._*	17.86	0.98	0.23	14.51
*E_Tens_*	12.61	0.99	0.18	10.12
*σ_Tens_*	49.14	0.88	4.63	35.03
*ɛ_Tens_*	30.34	0.94	0.612	23.65
*E_Flex_*	9.99	0.99	0.147	8.77
*σ_Flex_*	45.78	0.89	7.98	32.76
*E_IZOD_*	42.38	0.89	1.56	42.38
*E_Impact_*	43.23	0.89	0.64	32.59
Third-order polynomial equation				
*r* *_comp_*	5.20	0.99	0.01	3.32
*V_Vol._*	19.96	0.98	0.22	14.29
*E_Tens_*	16.35	0.99	0.19	10.63
*σ_Tens_*	30.93	0.96	2.63	19.91
*ɛ_Tens_*	39.84	0.95	0.57	22.15
*E_Flex_*	10.54	0.99	0.14	8.39
*σ_Flex_*	13.83	0.99	2.30	9.44
*E_IZOD_*	36.96	0.95	1.36	36.96
*E_Impact_*	31.08	0.95	0.42	21.45

**Table 5:** Regression coefficients.

Term	Coefficients								
	*ρ* *_comp_*	*V_Vol._*	*E_Tens_*	*σ_Tens_*	*ɛ_Tens_*	*E_Flex_*	*σ_Flex_*	*E_IZOD_*	*E_Impact_*
Constant	0.91	0.41	1.36680	28.9018	9.07400	1.38280	44.0908	2.03794	0.42030
*X*_1_	3.40 × 10^−3^	−3.68 × 10^−2^	0.08696	−0.53697	−0.29183	0.08468	−0.18528	0.07504	0.11780
*X*_2_	4.03 × 10^−3^	−2.05 × 10^−2^	0.08798	0.67296	0.07415	0.07744	1.07525	0.01891	0.15679
*X*_1_ *·* *X*_1_	1.25 × 10^−5^	1.19 × 10^−3^	-	0.05135	0.00260	-	0.06207	−0.00155	−0.00212
*X*_2_ *·* *X*_2_	-	2.15 × 10^−3^	-	0.04088	−0.00442	-	0.05684	0.00498	0.00003
*X*_1_ *·* *X*_2_	3.37 × 10^−5^	1.40 × 10^−3^	-	0.04161	0.00194	-	0.10456	0.02009	−0.00162
*X*_1_ *·* *X*_1_ *·* *X*_1_	-	-	-	−0.00079	-	-	−0.00109	−0.00001	-
*X*_1_ *·* *X*_1_ *·* *X*_2_	-	-	-	−0.00056	-	-	−0.00111	−0.00003	-
*X*_1_ *·* *X*_2_ *·* *X*_2_	-	-	-	−0.00111	-	-	−0.00239	−0.00026	-
*X*_2_ *·* *X*_2_ *·* *X*_2_	-	-	-	−0.00078	-	-	−0.00099	−0.00032	-

## Data Availability

The original contributions presented in this study are included in the article. Further inquiries can be directed to the corresponding author.

## Abbreviations

The following abbreviations are used in this manuscript:

WPC	Wood–polymer composites
NFC	Natural fibre composites
PP	Polypropylene
MA	Maleic anhydride
FFD	Full factorial design
